# 中国慢性淋巴细胞白血病/小淋巴细胞淋巴瘤的诊断与治疗指南（2025年版）

**DOI:** 10.3760/cma.j.cn121090-20241209-00551

**Published:** 2025-02

**Authors:** 中国抗癌协会血液肿瘤专业委员会 中华医学会血液学分会 中国慢性淋巴细胞白血病工作组

## Abstract

慢性淋巴细胞白血病（CLL）/小淋巴细胞淋巴瘤（SLL）是主要发生在中老年人群的、具有特殊免疫表型的成熟B淋巴细胞克隆增殖性肿瘤。近年来，CLL/SLL的基础与临床研究，特别是新药治疗领域取得了巨大进展。为提高我国医务工作者对CLL/SLL的诊断、鉴别诊断及规范化治疗水平，中国抗癌协会血液肿瘤专业委员会、中华医学会血液学分会和中国慢性淋巴细胞白血病工作组对中国CLL/SLL的诊断与治疗指南（2022年版）进行了修订，制订了本版指南。

## Abstract (en)

Chronic lymphocytic leukemia（CLL）/Small lymphocytic lymphoma（SLL）is a mature B cell neoplasm with specific immunophenotype, mainly occurs in the middle-aged and elderly population. In recent years, there has been significant progress in the basic and clinical research of CLL/SLL, especially in the field of new drug treatments. To improve the diagnostic, differential diagnostic, and standardized treatment levels of CLL/SLL for medical workers in China, Hematological Oncology Committee of China Anti-Cancer Association, Hematology Committee of Chinese Medical Association, Chinese Working Group for Chronic Lymphocytic Leukemia have revised the guidelines for the diagnosis and treatment of Chinese CLL/SLL（2022 edition）and formulated this version of the guidelines.

近年来，慢性淋巴细胞白血病（CLL）/小淋巴细胞淋巴瘤（SLL）的基础与临床研究，特别是新药治疗领域取得了巨大进展。为提高我国医务工作者对CLL/SLL的诊断、鉴别诊断及规范化治疗水平，中国抗癌协会血液肿瘤专业委员会、中华医学会血液学分会和中国慢性淋巴细胞白血病工作组组织相关专家对中国CLL/SLL的诊断与治疗指南（2022年版）[1]进行了修订，制订了本版指南。

一、定义

CLL/SLL是主要发生在中老年人群的一种具有特定免疫表型特点的成熟B淋巴细胞克隆增殖性肿瘤，以淋巴细胞在外周血、骨髓、脾和淋巴结聚集为特征。

二、诊断、分期、预后及鉴别诊断

1. 诊断：达到以下3项标准可以诊断：①外周血单克隆B淋巴细胞计数≥5×10^9^/L；②外周血涂片特征性地表现为小的、形态成熟的淋巴细胞显著增多，其细胞质少、核致密、核仁不明显、染色质部分聚集，并易见涂抹细胞；③外周血典型的流式细胞术免疫表型：CD5^+^、CD19^+^、CD23^+^、CD200^+^、ROR1^+^、CD10^-^、FMC7^-^、CD43^+^，表面免疫球蛋白（sIg）、CD20、CD22及CD79b的表达水平低于正常B细胞（dim）。流式细胞术确认B细胞的克隆性，即B细胞表面限制性表达κ或λ轻链（κ∶λ>3∶1或<0.3∶1）或>25％的B细胞sIg不表达[2]–[5]。SLL与CLL是同一种疾病的不同表现，淋巴组织具有CLL的细胞形态与免疫表型特征，确诊必须依赖病理组织学及免疫组化检查。临床特征：①淋巴结和（或）脾、肝肿大；②无血细胞减少；③外周血单克隆B淋巴细胞计数<5×10^9^/L。CLL与SLL的主要区别在于前者主要累及外周血和骨髓，而后者则主要累及淋巴结和骨髓。Lugano Ⅰ期SLL可局部放疗，其他SLL的治疗指征和治疗选择同CLL，以下均称为CLL。

单克隆B淋巴细胞增多症（MBL）：指健康个体外周血存在低水平的单克隆B淋巴细胞[4]–[6]。诊断标准：①B细胞克隆性异常；②外周血单克隆B淋巴细胞计数<5×10^9^/L；③无肝、肾、淋巴结肿大（淋巴结长径<1.5 cm）；④无骨髓浸润所致的贫血及血小板减少；⑤无慢性淋巴增殖性疾病（CLPD）的其他临床症状。根据免疫表型分为3种：CLL样表型、不典型CLL样表型和非CLL样表型。对于后两者需全面检查，如影像学、骨髓活检等，以排除外周血受累的非霍奇金淋巴瘤。对于CLL样表型MBL，需根据外周血克隆性B淋巴细胞计数分为“低计数”MBL（克隆性B淋巴细胞计数<0.5×10^9^/L）和“高计数”MBL（克隆性B淋巴细胞计数≥0.5×10^9^/L），“低计数”MBL无需常规临床随访，而“高计数”MBL的免疫表型、遗传学与分子生物学特征与Rai 0期CLL接近，需定期随访。

2. 分期及预后：CLL患者的中位生存期约10年，但不同患者的预后呈高度异质性。性别、年龄、体能状态、伴随疾病、外周血淋巴细胞计数及倍增时间、血清乳酸脱氢酶（LDH）、β_2_微球蛋白（β_2_-MG）、胸苷激酶1（TK1）等临床和实验室指标是重要的传统预后因素。临床上评估预后最常使用Rai和Binet两种临床分期系统（表1），这两种临床分期系统仅需体检和简单实验室检查，无需进行超声、CT或MRI等影像学检查。

**表1:** 慢性淋巴细胞白血病的临床分期系统

分期	定义
Binet分期	
A期	MBC≥5×10^9^/L，HGB≥100 g/L，PLT≥100×10^9^/L，<3个淋巴区域受累
B期	HGB≥100 g/L，PLT≥100×10^9^/L，≥3个淋巴区域受累
C期	HGB<100 g/L和（或）PLT<100×10^9^/L
Rai分期	
0期	仅MBC≥5×10^9^/L
Ⅰ期	MBC≥5×10^9^/L+淋巴结肿大
Ⅱ期	MBC≥5×10^9^/L+肝和（或）脾肿大±淋巴结肿大
Ⅲ期	HGB<110 g/L±淋巴结/肝/脾肿大
Ⅳ期	PLT<100×10^9^/L±淋巴结/肝/脾肿大

**注** 淋巴区域包括颈、腋下、腹股沟（单侧或双侧均计为1个区域）、肝和脾；MBC：单克隆B淋巴细胞计数；免疫性血细胞减少不作为分期的标准

然而上述两种临床分期系统存在以下缺陷：①处于同一分期的患者，其疾病发展过程存在异质性；②不能预测早期患者疾病是否进展及进展速度，而目前大多数患者诊断时处于疾病早期。目前预后意义比较明确的生物学标志有：免疫球蛋白重链可变区（IGHV）基因突变状态及片段使用，染色体异常［推荐CpG寡核苷酸+白细胞介素2（IL-2）刺激的染色体核型分析，荧光原位杂交（FISH）检测del（13q）、+12、del（11q）（ATM基因缺失）、del（17p）（TP53基因缺失）等］，基因突变［推荐二代基因测序检测TP53、NOTCH1（含非编码区）、SF3B1、BIRC3等基因］[7]–[8]。IGHV无突变的CLL患者预后较差；使用VH3-21的患者如属于B细胞受体（BCR）同型模式2亚群，则无论IGHV突变状态如何，预后均较差。具有染色体复杂核型异常、del（17p）和（或）TP53基因突变的患者预后最差，TP53基因或其他基因的亚克隆突变的预后价值有待进一步探讨，del（11q）是另一个预后不良标志。推荐应用CLL国际预后指数（CLL-IPI）进行综合预后评估[9]。CLL-IPI通过纳入TP53缺失和（或）突变、IGHV突变状态、β_2_-MG、临床分期、年龄，将CLL患者分为低危、中危、高危与极高危组（表2）。上述预后因素主要由接受化疗或化学免疫治疗患者获得，新药或新的治疗策略可能克服或部分克服上述不良预后。接受靶向治疗的患者的预后变量尚不明确。如果患者治疗后进展并有后续治疗的指征，在开始治疗前，应重新评估del（17p）或TP53突变状态、核型分析等。由于IGHV突变状态不会随时间改变，因此无需重复检测。

**表2:** 慢性淋巴细胞白血病国际预后指数（CLL-IPI）

参数	不良预后因素	积分	CLL-IPI积分	危险分层	5年生存率（％）
TP53异常	缺失或突变	4	0～1	低危	3.2
IGHV基因突变状态	无突变	2	2～3	中危	79.4
β_2_微球蛋白	>3.5 mg/L	2	4～6	高危	63.6
临床分期	Rai Ⅰ～Ⅳ期或BinetB～C期	1	7～10	极高危	23.3
年龄	>65岁	1			

**注** IGHV：免疫球蛋白重链可变区

3. 鉴别诊断：根据外周血淋巴细胞计数明显升高、典型的淋巴细胞形态及免疫表型特征，大多数CLL患者容易被诊断，但尚需与其他疾病，特别是其他B-CLPD相鉴别。根据CLL免疫表型积分系统（CD5^+^、CD23^+^、FMC7^-^、sIg^dim^、CD22/CD79b^dim/−^各积1分），CLL积分为4～5分，其他B-CLPD为0～2分。积分≤3分的患者需要结合淋巴结、脾脏、骨髓组织细胞学及遗传学、分子生物学检查等进行鉴别诊断，特别是套细胞淋巴瘤（MCL）、白血病期的边缘区淋巴瘤（尤其是脾边缘区淋巴瘤）、华氏巨球蛋白血症/淋巴浆细胞淋巴瘤（WM/LPL），它们也可表达CD5，但大多不表达CD23（特别是边缘区淋巴瘤）。具体参照《B细胞慢性淋巴增殖性疾病诊断与鉴别诊断中国专家共识（2018年版）》[10]。

三、治疗

（一）治疗指征

不是所有CLL都需要治疗，需具备以下至少1项方可进行治疗。

1. 进行性骨髓衰竭的证据：表现为血红蛋白和（或）血小板进行性减少。

2. 巨脾（如左肋缘下>6 cm）或有症状的脾肿大。

3. 巨块型淋巴结肿大（如最长直径>10 cm）或有症状的淋巴结肿大。

4. 进行性淋巴细胞增多，如2个月内淋巴细胞增多>50％，或淋巴细胞倍增时间（LDT）<6个月。如初始淋巴细胞计数<30×10^9^/L，不能单凭LDT作为治疗指征。

5. CLL/SLL导致的有症状的脏器功能异常（如皮肤、肾、肺、脊柱等）。

6. 自身免疫性溶血性贫血（AIHA）和（或）免疫性血小板减少症（ITP）对皮质类固醇反应不佳。

7. 至少存在下列一种疾病相关症状：①在前6个月内无明显原因的体重下降≥10％；②严重疲乏（如ECOG体能状态评分≥2分；不能进行常规活动）；③无感染证据，体温>38.0 °C，≥2周；④无感染证据，夜间盗汗>1个月。

8. 临床试验：符合所参加临床试验的入组条件。

不符合上述治疗指征的患者，每2～6个月随访1次，随访内容包括临床症状及体征，肝、脾、淋巴结肿大情况和血常规等。

（二）治疗前评估

治疗前（包括复发患者）必须对患者进行全面评估。评估内容包括：①病史和体格检查：特别是淋巴结（包括咽淋巴环和肝脾大小）；②体能状态：ECOG体能状态评分和（或）疾病累积评分表（CIRS）评分；③B症状：盗汗、发热、体重减轻；④血常规：包括白细胞计数及分类、血小板计数、血红蛋白等；⑤血清生化，包括肝肾功能、电解质、LDH等；⑥血清β_2_-MG；⑦骨髓活检±涂片：治疗前、疗效评估及鉴别血细胞减少原因时进行，典型病例的诊断、常规随访无需骨髓检查；⑧常规染色体核型分析；⑨检测del（13q）、+12、del（11q）、del（17p），TP53和IGHV等基因突变；⑩感染筛查：检测乙型肝炎病毒（HBV）、丙型肝炎病毒、人类免疫缺陷病毒、EB病毒等。

特殊情况下检测：免疫球蛋白定量；网织红细胞计数和直接抗人球蛋白试验（怀疑有溶血时必做）；心电图、超声心动图检查；如果计划进行全身治疗或放疗，育龄患者应进行妊娠检测和生育保护的讨论；颈、胸、腹、盆腔增强CT检查；PET-CT检查（怀疑Richter转化时）等。

（三）一线治疗选择[11]–[16]

根据TP53缺失和（或）突变、IGHV突变状态、年龄及身体状态进行分层治疗。患者的体能状态和实际年龄均为重要的参考因素，治疗前应评估患者的CIRS评分和身体适应性。因CLL目前仍为难以治愈性疾病，鼓励所有患者参加临床试验。

1. 无del（17p）/TP53基因突变CLL患者：

（1）身体状态良好（包括体力活动尚可、肌酐清除率≥70 ml/min及CIRS评分≤6分）的患者：优先推荐：泽布替尼、伊布替尼、FCR（氟达拉滨+环磷酰胺+利妥昔单抗）方案（用于IGHV基因突变且年龄<60岁的患者）、苯达莫司汀+利妥昔单抗（用于IGHV基因突变且年龄≥60岁的患者）。其他推荐：阿可替尼±奥妥珠单抗、奥布替尼、维奈克拉+奥妥珠单抗、布鲁顿酪氨酸激酶抑制剂（BTKi）联合FCR方案或FCG（氟达拉滨+环磷酰胺+奥妥珠单抗）方案（年龄<60岁）。

（2）身体状态欠佳的患者：优先推荐：泽布替尼、伊布替尼、苯丁酸氮芥+利妥昔单抗/奥妥珠单抗。其他推荐：阿可替尼±奥妥珠单抗、奥布替尼、维奈克拉+奥妥珠单抗、伊布替尼+维奈克拉、苯丁酸氮芥、利妥昔单抗。

2. 伴del（17p）/TP53基因突变CLL患者：

优先推荐：泽布替尼、伊布替尼。其他推荐：阿可替尼±奥妥珠单抗、奥布替尼、维奈克拉+奥妥珠单抗、伊布替尼+维奈克拉、大剂量甲泼尼龙+利妥昔单抗/奥妥珠单抗。

（四）复发、难治患者的治疗选择[17]–[21]

定义：复发：患者达到完全缓解（CR）或部分缓解（PR），≥6个月后疾病进展（PD）；难治：治疗失败（未获PR）或最后1次化疗后<6个月PD。复发、难治患者的治疗指征、治疗前检查同一线治疗（对于持续治疗中复发或PD的患者，无需具备治疗指征即可进行后线治疗），在选择治疗方案时除考虑患者的年龄、体能状态及遗传学等预后因素外，应同时综合考虑患者既往治疗方案的疗效（包括持续缓解时间）及耐受性等因素。BTKi或BCL2抑制剂（BCL2i）治疗无反应、PD的患者时，应分别检测BTK、PLCG2或BCL2突变状态，在没有PD的情况下，BTK、PLCG2或BCL2突变不是改变治疗方案的指征。

1. 无del（17p）/TP53基因突变CLL患者：

（1）身体状态良好的患者：优先推荐：泽布替尼、奥布替尼、阿可替尼、伊布替尼。其他推荐：FCR方案（年龄<60岁）、苯达莫司汀+利妥昔单抗、维奈克拉+利妥昔单抗/奥妥珠单抗（如果既往用作一线治疗并获得一段时间的缓解，优先使用维奈克拉+CD20单抗进行再治疗）、伊布替尼+维奈克拉、大剂量甲泼尼龙+利妥昔单抗、奥妥珠单抗、来那度胺±利妥昔单抗。

（2）身体状态欠佳的患者：优先推荐：泽布替尼、奥布替尼、阿可替尼、伊布替尼。其他推荐：苯丁酸氮芥+利妥昔单抗/奥妥珠单抗、伊布替尼+维奈克拉、维奈克拉+利妥昔单抗/奥妥珠单抗（如果既往用作一线治疗并获得一段时间的缓解，优先使用维奈克拉+CD20单抗进行再治疗）。

2. 伴del（17p）/TP53基因突变CLL患者：

优先推荐：泽布替尼、奥布替尼、阿可替尼、伊布替尼、维奈克拉+利妥昔单抗/奥妥珠单抗（如果既往用作一线治疗并获得一段时间的缓解，优先使用维奈克拉+奥妥珠单抗进行再治疗）。

3. BTKi和BCL2i治疗后的复发难治患者：

推荐：靶向CD19的嵌合抗原受体T（CAR-T）细胞治疗、PI3K抑制剂、伊布替尼+维奈克拉、大剂量甲泼尼龙+利妥昔单抗、来那度胺±利妥昔单抗、奥妥珠单抗、FCR方案（推荐用于≤60岁无合并症的患者）、苯达莫司汀+利妥昔单抗/奥妥珠单抗（用于>60岁患者或≤60岁有合并症的患者，不用于衰弱患者）。

对于使用BTKi持续治疗的患者，若出现不耐受的情况，可以考虑换用其他共价BTKi。

（五）维持治疗

应用BTKi单药治疗原则上需要持续治疗。如果患者因不能耐受、经济或其他原因需要停止治疗，建议在停药前桥接免疫化疗或BCL2i，以防疾病反弹。桥接治疗的疗程依据患者前期BTKi治疗的时间、缓解深度及耐受性等综合确定。

（六）新药治疗与新疗法

针对CLL的治疗药物开发获得快速发展，非共价BTKi匹妥布替尼（Pirtobrutinib）、Nemtabrutinib，PI3K抑制剂如林普利塞等正在临床探索中。此外，CAR-T细胞免疫疗法在复发/难治CLL临床试验中显示出较好疗效。BTKi与免疫化疗联合的有限周期治疗在CLL中取得高的微小残留病（MRD）阴性率和长期无进展生存率，值得尝试与探索，尤其对于年轻无TP53异常的患者[22]–[23]。

（七）造血干细胞移植

自体造血干细胞移植有可能改善患者的无进展生存，但并不延长总生存期，不推荐采用。异基因造血干细胞移植是CLL的治愈性手段，但由于CLL主要为老年患者，仅少数适合移植，近年来随着BTKi、BCL2i等小分子靶向药物的使用，异基因造血干细胞移植的地位和使用时机有所变化。适应证：对BTKi和BCL2i耐药的难治患者和CLL克隆相关Richter转化患者。

（八）组织学转化或进展的患者的治疗选择

对于临床上疑有转化的患者，应尽可能进行淋巴结切除活检明确诊断，当无法行切除活检时，可行粗针穿刺，结合免疫组化、流式细胞术等辅助检查明确诊断。PET-CT检查可用于指导活检部位（摄取最高部位）。

组织学转化在组织病理学上分为弥漫大B细胞淋巴瘤（DLBCL）与经典型霍奇金淋巴瘤（cHL）。对于前者，应进行CLL和转化后组织的IGHV基因测序以明确两者是否为同一克隆起源。

加速期CLL/SLL包括：①组织学侵袭型CLL/SLL：增殖中心扩张或融合（>20倍高倍视野）或高增殖指数（Ki-67>40％或每个增殖中心>2.4个有丝分裂象）；②幼淋进展型CLL：外周血或骨髓幼稚淋巴细胞比例增加（≥15％），通常伴有TP53异常，且排除MCL。

治疗前除进行常规CLL治疗前评估外，还需要进行PET-CT检查或增强CT检查。

1. Richter转化：对于Richter转化患者，需根据转化的组织学类型以及是否为克隆相关决定治疗方案。

（1）克隆无关的DLBCL：参照DLBCL进行治疗。

（2）克隆相关的DLBCL或不明克隆起源：可选用免疫化疗［R-DA-EPOCH、R-HyperCVAD（A方案）、R-CHOP方案］±维奈克拉[24]或±BTKi、PD-1单抗±BTKi、参加临床试验等方案，如取得缓解，尽可能进行异基因造血干细胞移植，否则参照难治复发DLBCL治疗方案。

（3）cHL：参考cHL治疗方案。

2. 加速期CLL：加速期CLL不同于Richter转化，但预后较差，最佳的治疗方案尚未明确。临床实践中，参照CLL治疗方案。

（九）支持治疗

1. 感染预防：对于反复感染且IgG<5 g/L的CLL患者，需进行静脉注射丙种球蛋白（IVIG）至IgG>5 g/L以提高机体非特异性免疫力。

2. 对于使用BTKi、嘌呤类似物或苯达莫司汀为基础的免疫化疗、维奈克拉或PI3K抑制剂治疗的患者，需预防疱疹病毒感染、肺孢子菌肺炎（PJP），必须密切监测包括HBV和巨细胞病毒等病毒指标。HBV再激活：参照《中国淋巴瘤合并HBV感染患者管理专家共识》[25]进行预防和治疗。新型冠状病毒防治：参照《惰性B细胞非霍奇金淋巴瘤患者新型冠状病毒感染防治专家共识》[26]。

3. 免疫性血细胞减少：

（1）糖皮质激素是一线治疗，无效的患者可选择行IVIG、利妥昔单抗、环孢素A及脾切除等治疗。

（2）氟达拉滨相关的自身免疫性溶血，应停止使用并避免再次使用。

4. 肿瘤溶解综合征（TLS）：对于TLS发生风险较高的患者，应密切监测相关血液指标（钾、尿酸、钙、磷及LDH等），同时进行充足的水化碱化。采用维奈克拉治疗的患者应进行TLS危险分级并予以相应的预防措施。

四、疗效标准

在CLL患者的治疗中应定期进行疗效评估，BTKi单药持续治疗通常以6个疗程为宜，联合免疫化疗或包含新药的固定周期患者建议每2～3个疗程进行疗效评价。疗效标准[5]见表3。CR：达到表3所有标准，无疾病相关症状；骨髓未恢复的CR（CRi）：除骨髓未恢复正常外，其他符合CR标准；PR：至少达到2个A组标准+1个B组标准；疾病稳定（SD）：疾病无进展同时未达到PR；PD：达到任何1个A组或B组标准；复发：患者达到CR或PR，≥6个月后PD；难治：治疗失败（未获CR或PR）或最后1次化疗后<6个月PD；伴有淋巴细胞计数升高的PR（PR-L）：BCR信号通路的小分子抑制剂如BTKi和PI3K抑制剂治疗后出现短暂淋巴细胞计数升高，淋巴结、脾脏缩小，淋巴细胞计数升高在最初几周出现，并会持续数月，此时单纯的淋巴细胞计数升高不作为PD；MRD阴性：指残存白血病细胞<1×10^−4^。临床试验证据表明，固定疗程治疗结束后，外周血MRD阴性是疗效的重要预测指标，但目前不作为疗效评价标准。等位基因特异性寡核苷酸聚合酶链反应（ASO-PCR）和八色流式细胞术是用于10^−4^至10^−5^水平MRD检测的两种验证方法。基于二代测序的检测方法已被证明更敏感，可以在10^−6^水平检测MRD，具体参照《慢性淋巴细胞白血病微小残留病检测与临床解读中国专家共识（2023年版）》[27]。初步疗效评估为CR的患者应进行骨髓穿刺及活检检查。骨髓检查时机：化疗或化学免疫治疗方案结束后至少2个月；BTKi需要持续治疗的患者，应在患者达到最佳反应至少2个月后。骨髓活检是确认CR的必要检查，对于其他条件符合CR而免疫组织化学显示存在CLL细胞组成的淋巴小结的患者，评估为结节性部分缓解（nPR）。SLL疗效评估参照2014 Lugano淋巴瘤疗效评估标准[28]。

**表3:** 慢性淋巴细胞白血病（CLL）的疗效标准

参数	CR	PR	PR-L	PD
A组：评价肿瘤负荷				
淋巴结肿大	无>1.5 cm	缩小≥50％	缩小≥50％	增大≥50％
肝肿大	无	缩小≥50％	缩小≥50％	增大≥50％
脾肿大	无	缩小≥50％	缩小≥50％	增大≥50％
骨髓	增生正常，淋巴细胞比例<30％，无B细胞性淋巴小结；骨髓增生低下则为CR伴骨髓造血不完全恢复<4×10^9^/L	骨髓浸润较基线降低≥50％ 或出现B细胞性淋巴小结	骨髓浸润较基线降低≥50％ 或出现B细胞性淋巴小结	–
ALC	<4×10^9^/L	较基线降低≥50％	淋巴细胞升高	较基线升高≥50％
B组：评价骨髓造血功能				
PLT（不使用生长因子）	>100×10^9^/L	>100×10^9^/L或较基线升高≥50％	>100×10^9^/L或较基线升高≥50％	CLL本病所致下降≥50％
HGB（无输血、不使用生长因子）	>110 g/L	–	>110 g/L或较基线升高≥50％	CLL本病所致下降>20 g/L
ANC（不使用生长因子）	>1.5×10^9^/L	>1.5×10^9^/L或较基线升高>50％	>1.5×10^9^/L或较基线升高>50％	–

**注** ALC：外周血淋巴细胞绝对计数；ANC：外周血中性粒细胞绝对计数；CR：完全缓解；PR：部分缓解；PR-L：伴有淋巴细胞增高的PR；PD：疾病进展；–：未作要求

五、随访

完成免疫化疗或包含新药的固定周期治疗达CR或PR的患者，应该定期进行随访，包括每3个月血细胞计数及肝、脾、淋巴结触诊检查等。由于BTKi持续治疗需要长期使用至PD或不能耐受，因此患者在BTKi治疗期间应定期进行随访，包括每1～3个月行血细胞计数，肝、脾、淋巴结触诊检查及BTKi相关不良反应监测等，针对BTKi相关的心血管疾病合并症，参照《布鲁顿酪氨酸激酶抑制剂治疗B细胞淋巴瘤相关心血管疾病合并症管理专家共识（2023年版）》[29]。此外还应特别注意第二肿瘤的出现。
